# Knowledge, attitudes, and practices of healthcare professionals regarding neuropathic pain in spinal cord injury in Hunan, China

**DOI:** 10.1038/s41598-025-16252-6

**Published:** 2025-08-20

**Authors:** Lan Luo, Zhenhua Ming, Yuliang Huang, Lifang Huang, Jiawei Cao

**Affiliations:** 1 Department of Rehabilitation, Hunan Provincial People’s Hospital (The First Affiliated Hospital of Hunan Normal University), No. 61 Jiefang West Road, Furong District, Changsha, 410021 Hunan province China; 2 Department of Anesthesiology, Changsha Central Hospital, University of South China, 161 Shaoshan South Road, Changsha, Changsha, 410028 Hunan China

**Keywords:** Spinal cord injury, Neuropathic pain, Health knowledge, attitudes, practice, Healthcare providers, Cross-sectional study, Neuroscience, Diseases

## Abstract

To evaluate healthcare professionals’ knowledge, attitude, and practice regarding spinal cord injury neuropathic pain in Hunan Province, China. This cross-sectional study was conducted between August 2024 and September 2024 in Hunan Province and included healthcare professionals. The questionnaire was self-designed (Cronbach’s α = 0.954) to collect demographic and KAP data. A structural equation modeling (SEM) analysis was performed to determine the relationships among KAP dimensions. The analysis included 402 valid questionnaires. Among them, 28.1% were working in the rehabilitation department, 62.2% had experience in treating patients with SCI, 59.5% had experience in treating patients with SCI-NP, 79.6% were working in a public tertiary hospital, and 85.1% were working at a teaching hospital. The mean knowledge, attitude, and practice scores were 13.02 ± 8.24 (/28, 46.5%), 34.80 ± 5.99 (/40, 87.0%), and 21.56 ± 8.03 (/35, 61.6%), indicating poor knowledge, positive attitudes, and moderate practice, respectively. SEM analysis indicated knowledge directly influenced attitudes (β = 0.511, *P* < 0.001) and practices (β = 0.571, *P* < 0.001) and indirectly influenced practice (β = 0.093, *P* < 0.001), while attitudes directly influenced practice (β = 0.181, *P* < 0.001). Similar relationships were observed in physicians and nurses. Healthcare professionals exhibit poor knowledge, positive attitudes, and moderate practice regarding SCI-NP. Educational initiatives are needed to enhance their KAP, translating improved knowledge and attitudes into better practices.

**Supplementary Information:**

The online version contains supplementary material available at 10.1038/s41598-025-16252-6.

## Introduction

Spinal cord injury (SCI) is damage to the spinal cord, most often due to physical trauma, and leading to transient or permanent loss of motor, sensory, and autonomic functions^1^. The common causes include motor vehicle accidents, falls, gunshot wounds and other forms of violence, and sports and recreational activities^2^. The annual incidence of SCI varies from 15 to 52.5 cases per million persons^3^. Beyond the important neurological deficit, persons with SCI may develop numerous multisystem complications during the acute phase, including brain neurodegeneration^4^ and cognitive impairment^5^.

Chronic pain (including visceral, musculoskeletal, and neuropathic) is reported in up to 94% of persons with SCI^6^. Neuropathic pain (NP) is due to damage to the somatosensory nervous system itself, causing pain signals, as opposed to communicating damage from non-neural tissue^7^. The patient-reported descriptions of SCI-related NP (SCI-NP) include burning, tingling, pricking, pins and needles, sharp, electric, stinging, pressing, shooting, squeezing, stabbing, painful cold, and electric-shock-like. There may be sensory dysfunctions such as hypoesthesia, allodynia, and hyperalgesia. NP within 1 year of the injury is reported in 40%−50% of patients; pain developing > 1 year after injury can be due to other causes^8^. SCI-NP can have profound impacts on functions, activity, sleep, mood, and the use of medications such as opioids or substances^9^. Therefore, it is recommended to regularly evaluate the patient with SCI for pain, including NP, based on complete patient history and physical examination because the prognosis of NP is better if it is managed early since NP can progress to chronic, refractory pain, which is often more difficult to manage^10,11^. The optimal management strategy is based on identifying the underlying conditions that cause the pain, aggravate the pain, and mimic NP characteristics. The strategy also include managing the psychosocial factors that may contribute to distress and disability related to the pain, and addressing pain-related issues with activity, sleep, mood, and medications^9^. The management of NP includes patient education, cognitive behavioral therapy, self-management, group discussions, exercise, and medication^9^.

Proper knowledge and attitudes are required to identify, assess, and manage NP in patients with SCI. Identifying the gaps, misunderstandings, and misconceptions could help design and implement educational interventions that could translate into better practice toward SCI-NP. Knowledge, attitude, and practice (KAP) studies allow the identification of such barriers to the optimal performance of a given healthcare concept^12,13^. Although several studies have examined the KAP of physicians toward chronic pain^14–16^, to our knowledge none have examined the KAP of healthcare professionals toward SCI-NP.

Therefore, this study aimed to examine the KAP of healthcare professionals toward SCI-NP.

## Methods

### Study design and subjects

This cross-sectional study was conducted in Hunan Province between August 2024 and September 2024. The study enrolled healthcare professionals. The study was approved by the Medical Ethics Committee of Hunan Provincial People’s Hospital (2024 − 304) and Changsha Central Hospital Medical (2024 − 106). All participants provided informed consent before completing the questionnaire.

The inclusion criteria were (1) board-certified healthcare professionals with full-time employment and (2) voluntary participation. The exclusion criteria were 1) < 1 year of employment or 2) on leave.

### Questionnaire

The design of the questionnaire was based on guidelines related to SCI-NP: the “Chinese Guidelines for the Assessment and Management of Neuropathic Pain (2024 Edition)” and “The CanPain SCI Clinical Practice Guidelines for Rehabilitation Management of Neuropathic Pain After Spinal Cord Injury: 2021 Update”^9^. The questionnaire was pre-tested with a small sample (28 individuals), yielding a reliability coefficient of 0.954. During the pilot study, participants were asked whether they found any questions difficult to answer. Since no one reported any difficulties, the questionnaire was used in the subsequent study.

The final questionnaire was in Chinese and included four sections: demographic information, knowledge dimension, attitude dimension, and practice dimension. The knowledge dimension consisted of 14 questions. Questions 1–4, 8, 9, 12, 13, and 14 had four items each; “know” was scored 2 points, “partially know” was 1 point, and “do not know” was 0 points. For questions 5, 6, 7, 10, and 11, the final score was calculated by summing the sub-items and dividing by the number of sub-items, then multiplying by 2. The total knowledge score ranged 0–28. The attitude dimension included eight questions using a five-point Likert scale, from strongly agree (5 points) to strongly disagree (1 point), with a total score range of 8–40. The practice dimension included eight questions; questions 1–7 used a 5-point Likert scale from always (5 points) to never (1 point), and question 8 was analyzed descriptively. The practice score ranged 7–35.

Patients’ overall KAP dimension scores were categorized using a modified Bloom’s criteria cutoff point: respondents who scored between 80% and 100% were considered to have good knowledge, positive attitude, and appropriate practice, respectively, 60%−79% as moderate, and < 60% as poor knowledge, negative attitude, and inappropriate practice, respectively^17,18^.

### Questionnaire deployment

Questionnaires were distributed to the participants through quick response (QR) codes distributed to the potential participants by the heads of the human resource departments of the selected hospitals through professional WeChat groups of verified healthcare professionals. Reading the QR code granted access to the online questionnaire. The participants were enrolled through convenience sampling. The first page was the informed consent form. Clicking “I agree to participate in this study” was mandatory to gain access to the questionnaire. Responses to all items were mandatory to submit the questionnaire. A given IP address could be used only once to submit a questionnaire. Questionnaires responded in < 60 s (43 questions at about 1.5 s each) or in > 10 min were considered invalid and were excluded. The lower time limit was set to ensure the participants read the items before answering them. The upper time limit was set to ensure the participants did not take time to look for answers on the Internet or discuss with colleagues. The upper limit also ensured that the participant did not pause answering and answering the two parts in different sets of minds. Questionnaires responded with an obvious pattern (e.g., all first choices) were also considered invalid.

### Sample size

The minimal sample for this study was calculated based on Cochran’s sample size estimation equation (n = z^2^pq/e^2^)^19^. In this formula, n is the number of participants required, z = 1.96 in 95% confidence interval, p = expected proportion, q = 1-p, and e is 5% of the margin of error. Since no previous studies reported the KAP toward SCI-NP in this population, 50% was selected as the expected proportion to maximize the sample size. Applying the values mentioned above in Cochran’s equation, the estimated sample size for the present study was 384.

### Statistical analysis

The continuous data were tested for normality using the Kolmogorov-Smirnov test. Normally distributed data were presented as means ± standard deviations and analyzed using Student’s t-test (two-level comparisons) or ANOVA (multilevel comparisons). Non-normally distributed data were presented as medians (interquartile range (IQR)) and were analyzed using the Mann-Whitney U-test (two-level comparisons) or the Kruskal-Wallis H-test (multilevel comparisons). Categorical data were presented as n (%). Pearson’s correlation analysis was used to correlate dimension scores when normally distributed data. When the data were not normally distributed, Spearman’s correlation analysis was used. SPSS 23.0 (IBM, Armonk, NY, USA) was used for analysis. Two-sided P-values < 0.05 were considered statistically significant.

Based on the KAP theoretical framework, structural equation modeling (SEM) was used to test three hypotheses: H1) knowledge influences attitudes, H2: attitudes influence practice, and H3) knowledge influences practice). The direct and indirect effects were calculated. Stata 18.0 (StataCorp LP, College Station, TX, USA) was used for the SEM analysis.

## Results

### Characteristics of the participants

In this study, 402 valid questionnaires were included for analysis. The characteristics of the participants are shown in Table 1. The majority of the participants were 29–35 years old (42.3%), female (75.6%), with a bachelor’s degree (69.9%), nurses (62.7%), with an intermediate professional title (56.2%), with 6–10 years of experience (54.2%), working in the rehabilitation department (28.1%), with experience in treating patients with SCI (62.2%), with experience in treating patients with SCI-NP (59.5%), working in a public tertiary hospital (79.6%), and working at a teaching hospital (85.1%).

**Table 1:** Characteristics of the participants.

*N* = 402	*n* (%)	Knowledge	*P*	Attitude	*P*	Practice	*P*
		Mean ± SD		Mean ± SD		Mean ± SD	
	402 (100.0)	13.02 ± 8.24		34.80 ± 5.99		21.56 ± 8.03	
Age (years)			0.443		0.238		0.744
*≤* 28	94 (23.4)	12.16 ± 8.52		34.05 ± 6.00		21.29 ± 7.93	
29–35	170 (42.3)	13.61 ± 7.97		35.16 ± 5.92		21.38 ± 7.83	
*≥* 35	138 (34.3)	12.88 ± 8.37		34.87 ± 6.05		21.97 ± 8.36	
Gender			0.735		0.321		0.610
Male	98 (24.4)	13.23 ± 8.41		33.98 ± 6.90		21.31 ± 7.78	
Female	304 (75.6)	12.95 ± 8.20		35.07 ± 5.65		21.64 ± 8.11	
Education level			0.188		0.168		0.006
Associate degree or below	33 (8.2)	15.63 ± 8.66		34.58 ± 5.08		25.85 ± 8.43	
Bachelor’s degree	281 (69.9)	12.72 ± 8.15		34.48 ± 6.24		21.15 ± 7.86	
Master’s degree or above	88 (21.9)	13.01 ± 8.28		35.91 ± 5.35		21.25 ± 8.01	
Profession			0.921		0.567		0.044
Physician	137 (34.1)	12.83 ± 8.04		34.82 ± 6.48		20.37 ± 7.97	
Nurse	252 (62.7)	13.08 ± 8.38		34.73 ± 5.72		22.06 ± 7.94	
Rehabilitation therapist	13 (3.2)	13.79 ± 8.15		36.00 ± 6.01		24.31 ± 9.10	
Professional title			0.023		0.049		0.788
Junior	109 (27.1)	13.68 ± 8.26		34.23 ± 6.16		21.54 ± 8.22	
Intermediate	226 (56.2)	12.46 ± 8.17		34.58 ± 6.14		21.34 ± 7.91	
Senior	41 (10.2)	15.93 ± 7.76		36.95 ± 4.46		22.44 ± 8.33	
No title	26 (6.5)	10.59 ± 8.47		35.73 ± 5.43		22.15 ± 8.03	
Work experience (years)			0.924		0.803		0.806
*≤* 5	105 (26.1)	13.12 ± 8.38		34.62 ± 6.16		21.13 ± 7.69	
6–10	218 (54.2)	12.93 ± 8.16		34.71 ± 6.16		21.63 ± 8.20	
> 10	79 (19.7)	13.13 ± 8.37		35.29 ± 5.27		21.92 ± 8.04	
Department			< 0.001		< 0.001		< 0.001
Rehabilitation	113 (28.1)	16.77 ± 7.71		36.65 ± 5.01		25.30 ± 7.17	
Anesthesiology	88 (21.9)	14.45 ± 7.14		35.68 ± 5.28		22.08 ± 7.25	
Pain management	4 (1.0)	15.70 ± 6.76		32.00 ± 7.30		23.75 ± 5.91	
Spinal Surgery	7 (1.7)	17.29 ± 5.80		37.57 ± 3.55		24.00 ± 6.81	
Neurosurgery	11 (2.7)	15.71 ± 8.29		36.91 ± 3.73		24.09 ± 6.52	
Other	179 (44.5)	9.56 ± 7.84		33.03 ± 6.54		18.64 ± 8.01	
Experience with patients with spinal cord injury?			< 0.001		< 0.001		< 0.001
Yes	250 (62.2)	15.31 ± 7.91		36.10 ± 4.78		23.45 ± 7.33	
No	152 (37.8)	9.25 ± 7.35		32.66 ± 7.08		18.45 ± 8.17	
Average number of spinal cord injury patients treated/cared for per month in the past year			< 0.001		0.026		< 0.001
<1	238 (59.2)	10.58 ± 7.90		34.03 ± 6.55		19.66 ± 8.15	
1–5	115 (28.6)	16.04 ± 7.42		35.83 ± 4.69		24.43 ± 6.37	
6–10	32 (8.0)	17.25 ± 7.29		35.31 ± 5.53		23.50 ± 7.53	
> 10	17 (4.2)	18.84 ± 7.47		37.59 ± 4.58		25.12 ± 9.96	
Patients with neuropathic pain among the spinal cord injury patients treated or cared for			< 0.001		0.004		< 0.001
Yes	239 (59.5)	15.69 ± 7.73		35.72 ± 4.92		23.84 ± 7.24	
No	163 (40.5)	9.10 ± 7.36		33.45 ± 7.07		18.22 ± 7.97	
The average number of spinal cord injury neuropathic pain patients treated/cared for per month in the past year			< 0.001		0.09		< 0.001
< 1	264 (65.7)	10.78 ± 7.77		34.28 ± 6.40		19.89 ± 7.83	
1–5	108 (26.9)	16.73 ± 7.26		35.60 ± 4.97		24.00 ± 7.19	
*≥* 6	30 (7.5)	19.41 ± 7.56		36.53 ± 5.02		27.47 ± 7.80	
Type of hospital			0.612		0.581		0.288
Public tertiary hospital	320 (79.6)	13.13 ± 8.19		34.91 ± 5.88		21.40 ± 7.75	
Other	82 (20.4)	12.61 ± 8.44		34.39 ± 6.41		22.18 ± 9.06	
Teaching hospital			0.538		0.733		0.463
Yes	342 (85.1)	13.14 ± 8.20		34.79 ± 5.90		21.70 ± 7.89	
No	60 (14.9)	12.37 ± 8.51		34.85 ± 6.52		20.75 ± 8.81	

SD: standard deviation.

### Knowledge, attitudes, and practices

The mean knowledge, attitude, and practice scores were 13.02 ± 8.24 (/28, 46.5%), 34.80 ± 5.99 (/40, 87.0%), and 21.56 ± 8.03 (/35, 61.6%), indicating poor knowledge, positive attitudes, and moderate practice. The knowledge scores were associated with the professional title (*P* = 0.023), department (*P* < 0.001), experience with treating SCI (*P* < 0.001), the number of patients with SCI treated per month (*P* < 0.001), experience with treating SCI-NP (*P* < 0.001), and the number of patients with SCI-NP treated per month (*P* < 0.001). The attitude scores were associated with the professional title (*P* = 0.049), department (*P* < 0.001), experience with treating SCI (*P* < 0.001), the number of patients with SCI treated per month (*P* = 0.026), and experience with treating SCI-NP (*P* = 0.004). The practice score was associated with education (*P* = 0.006), profession (*P* = 0.044), department (*P* < 0.001), experience with treating SCI (*P* < 0.001), the number of patients with SCI treated per month (*P* < 0.001), experience with treating SCI-NP (*P* < 0.001), and the number of patients with SCI-NP treated per month (*P* < 0.001) (Table 1).

In the knowledge dimension, the items with the poorest knowledge were K5, K14, and K4 (Table 2). The lowest proportions of “strongly agree” and “agree” were observed for A1, A5, and A7 (Table 3). The lowest proportions of “always” and “often” for the practice items were observed for P2, P1, and P3 (Table 4).

**Table 2:** Knowledge dimension distribution.

Knowledge	Know	Partially know	Do not know
K1. Pain is a common complication after SCI.	165 (41%)	182 (45.3%)	55 (13.7%)
K2. NP refers to pain caused by damage or disease affecting the somatosensory system.	130 (32.3%)	203 (50.5%)	69 (17.2%)
K3. Pain screening for SCI patients should be conducted continuously during admission, hospitalization, and follow-up after discharge.	128 (31.8%)	174 (43.3%)	100 (24.9%)
K4. Are you familiar with the International Spinal Cord Injury Pain Basic Data Set (ISCIPBDS)?	52 (12.9%)	138 (34.3%)	212 (52.7%)
K5. All SCI patients should undergo pain assessment. The following scales can be used for NP assessment:			
K5.1 DN4 and I-DN4	125 (31.1%)	11 (2.7%)	266 (66.2%)
K5.2 LANSS and S-LANSS	87 (21.6%)	21 (5.2%)	294 (73.1%)
K5.3 PainDETECT	105 (26.1%)	12 (3%)	285 (70.9%)
K5.4 NPQ	100 (24.9%)	13 (3.2%)	289 (71.9%)
K5.5 ID pain	117 (29.1%)	10 (2.5%)	275 (68.4%)
K6. Electrophysiological tests play an important role in the diagnosis of NP. The following tests are helpful for NP assessment:			
K6.1 Nerve conduction studies	263 (65.4%)	2 (0.5%)	137 (34.1%)
K6.2 F-waves and H-reflexes	161 (40%)	15 (3.7%)	226 (56.2%)
K6.3 Quantitative sensory testing	202 (50.2%)	10 (2.5%)	190 (47.3%)
K6.4 Skin sympathetic response	205 (51%)	15 (3.7%)	182 (45.3%)
K7. The following imaging studies help diagnose NP:			
K7.1 Computed tomography	205 (51%)	48 (11.9%)	149 (37.1%)
K7.2 Magnetic resonance imaging	260 (64.7%)	13 (3.2%)	129 (32.1%)
K7.3 Functional magnetic resonance imaging	196 (48.8%)	17 (4.2%)	189 (47%)
K7.4 Positron emission tomography-computed tomography	177 (44%)	50 (12.4%)	175 (43.5%)
K8. Pain is related to psychological states such as anxiety and depression. PHQ-9 and GAD-7 are commonly used for psychological assessment in NP patients.	109 (27.1%)	140 (34.8%)	153 (38.1%)
K9. Management of NP requires a principle of comprehensive management and multidisciplinary collaboration.	135 (33.6%)	136 (33.8%)	131 (32.6%)
K10. First-line medications for NP include:			
K10.1 Anticonvulsants	167 (41.5%)	41 (10.2%)	194 (48.3%)
K10.2 Antidepressants	178 (44.3%)	37 (9.2%)	187 (46.5%)
K10.3 Opioids	209 (52%)	37 (9.2%)	156 (38.8%)
K10.4 Topical analgesics	246 (61.2%)	20 (5%)	136 (33.8%)
K11. Minimally invasive interventional treatments for NP include:			
K11.1 Nerve blocks	250 (62.2%)	22 (5.5%)	130 (32.3%)
K11.2 Pulsed radiofrequency	234 (58.2%)	18 (4.5%)	150 (37.3%)
K11.3 Neurolysin	196 (48.8%)	32 (8%)	174 (43.3%)
K12. Neuromodulation treatments for NP include neurostimulation and intrathecal drug delivery.	109 (27.1%)	127 (31.6%)	166 (41.3%)
K13. Surgical treatments for NP mainly involve nerve decompression and neurodestruction.	100 (24.9%)	137 (34.1%)	165 (41%)
K14. Are you familiar with the following NP treatments:			
K14.1 Psychotherapy	108 (26.9%)	157 (39.1%)	137 (34.1%)
K14.2 Rehabilitation therapy	151 (37.6%)	155 (38.6%)	96 (23.9%)
K14.3 Physical therapy	144 (35.8%)	154 (38.3%)	104 (25.9%)
K14.4 Gene therapy	77 (19.2%)	104 (25.9%)	221 (55%)

SCI: spinal cord injury; NP: neuropathic pain.

Note: The questionnaire was designed and administered in Chinese. The English translation was not validated and is provided for indicative purposes only.

**Table 3:** Attitude dimension distribution.

Attitude	Strongly agree	Agree	Neutral	Disagree	Strongly disagree
A1. NP has a significant impact on SCI patients’ emotions, quality of life, and rehabilitation.	201 (50%)	131 (32.6%)	59 (14.7%)	4 (1%)	7 (1.7%)
A2. Early detection and treatment of NP are important for the rehabilitation of SCI patients.	219 (54.5%)	127 (31.6%)	51 (12.7%)	1 (0.2%)	4 (1%)
A3. When assessing NP, the patient’s concerns, expectations, and needs should be considered.	209 (52%)	136 (33.8%)	52 (12.9%)	0 (0%)	5 (1.2%)
A4. The assessment and management of SCI-NP require multidisciplinary collaboration.	214 (53.2%)	131 (32.6%)	54 (13.4%)	0 (0%)	3 (0.7%)
A5. Self-management by patients plays an important role in the management of SCI-NP.	210 (52.2%)	127 (31.6%)	60 (14.9%)	1 (0.2%)	4 (1%)
A6. Treatment of SCI-NP should consider both pharmacological and non-pharmacological treatment options.	217 (54%)	127 (31.6%)	52 (12.9%)	2 (0.5%)	4 (1%)
A7. Management of SCI-NP should continue after the patient is discharged.	217 (54%)	122 (30.3%)	55 (13.7%)	0 (0%)	8 (2%)
A8. Before the discharge of SCI-NP patients, a comprehensive follow-up rehabilitation plan should be developed, and self-management skills should be taught to the patients.	215 (53.5%)	126 (31.3%)	54 (13.4%)	3 (0.7%)	4 (1%)

SCI: spinal cord injury; NP: neuropathic pain.

Note: The questionnaire was designed and administered in Chinese. The English translation was not validated and is provided for indicative purposes only.

**Table 4:** Practice dimension distribution.

Practice	Always	Often	Sometimes	Rarely	Never
P1. I actively learn about the management of SCI-NP.	56 (13.9%)	50 (12.4%)	99 (24.6%)	123 (30.6%)	74 (18.4%)
P2. I actively learn about the latest developments in SCI-NP or NP research.	52 (12.9%)	47 (11.7%)	91 (22.6%)	124 (30.8%)	88 (21.9%)
P3. During the management of SCI patients, I follow guidelines for pain assessment.	74 (18.4%)	72 (17.9%)	90 (22.4%)	99 (24.6%)	67 (16.7%)
P4. When managing SCI-NP patients, I first provide education to the patients.	87 (21.6%)	89 (22.1%)	74 (18.4%)	95 (23.6%)	57 (14.2%)
P5. When using medication for treatment, I strictly adhere to guidelines or recommendations.	142 (35.3%)	89 (22.1%)	64 (15.9%)	63 (15.7%)	44 (10.9%)
P6. Before SCI-NP patients are discharged, I provide them with a long-term rehabilitation plan.	102 (25.4%)	83 (20.6%)	84 (20.9%)	81 (20.1%)	52 (12.9%)
P7. Before SCI-NP patients are discharged, I provide education on self-management.	110 (27.4%)	81 (20.1%)	81 (20.1%)	77 (19.2%)	53 (13.2%)
P8.1 Cognitive behavioral therapy	137 (34.1%)	152 (37.8%)	68 (16.9%)	8 (2%)	37 (9.2%)
P8.2 Hyperbaric oxygen therapy	103 (25.6%)	159 (39.6%)	90 (22.4%)	13 (3.2%)	37 (9.2%)
P8.3 Anti-inflammatory diet	92 (22.9%)	149 (37.1%)	98 (24.4%)	15 (3.7%)	48 (11.9%)
P8.4 Respiratory control electrical stimulation	92 (22.9%)	147 (36.6%)	87 (21.6%)	5 (1.2%)	71 (17.7%)
P8.5 Neurolysin	77 (19.2%)	142 (35.3%)	94 (23.4%)	5 (1.2%)	84 (20.9%)
P8.6 Autologous mesenchymal stem cell transplantation	64 (15.9%)	122 (30.3%)	90 (22.4%)	13 (3.2%)	113 (28.1%)
P8.7 Meditation	63 (15.7%)	148 (36.8%)	109 (27.1%)	22 (5.5%)	60 (14.9%)
P8.8 Hypnosis	66 (16.4%)	145 (36.1%)	124 (30.8%)	20 (5%)	47 (11.7%)
P8.9 Acupuncture	118 (29.4%)	194 (48.3%)	62 (15.4%)	4 (1%)	24 (6%)
P8.10 Exercise	113 (28.1%)	170 (42.3%)	79 (19.7%)	15 (3.7%)	25 (6.2%)
P8.11 Osteopathy	55 (13.7%)	121 (30.1%)	129 (32.1%)	29 (7.2%)	68 (16.9%)

SCI: spinal cord injury; NP: neuropathic pain.

Note: The questionnaire was designed and administered in Chinese. The English translation was not validated and is provided for indicative purposes only.

### Correlations

In all participants, the knowledge scores were correlated to the attitude (*r* = 0.477, *P* < 0.001) and practice (*r* = 0.646, *P* < 0.001) scores, while the attitude scores were correlated to the practice scores (*r* = 0.428, *P* < 0.001). In physicians, the knowledge scores were correlated to the attitude (*r* = 0.527, *P* < 0.001) and practice (*r* = 0.637, *P* < 0.001) scores, while the attitude scores were correlated to the practice scores (*r* = 0.468, *P* < 0.001). In nurses, the knowledge scores were correlated to the attitude (*r* = 0.454, *P* < 0.001) and practice (*r* = 0.645, *P* < 0.001) scores, while the attitude scores were correlated to the practice scores (*r* = 0.413, *P* < 0.001) (Supplementary Table S1).

### Structural equation modeling analysis

In all participants, knowledge directly influenced attitudes (β = 0.511, *P* < 0.001) and practices (β = 0.571, *P* < 0.001) and indirectly (through attitude) influenced practice (β = 0.093, *P* < 0.001), while attitudes directly influenced practice (β = 0.181, *P* < 0.001). In physicians, knowledge directly influenced attitudes (β = 0.580, *P* < 0.001) and practices (β = 0.563, *P* < 0.001) and indirectly (through attitude) influenced practice (β = 0.110, *P* = 0.025), while attitudes directly influenced practice (β = 0.189, *P* = 0.022). In nurses, knowledge directly influenced attitudes (β = 0.483, *P* < 0.001) and practices (β = 0.564, *P* < 0.001) and indirectly (through attitude) influenced practice (β = 0.085, *P* = 0.005), while attitudes directly influenced practice (β = 0.175, *P* = 0.004) (Table 5).

**Table 5:** SEM.

Model paths		Total effects		Direct effect		Indirect effect	
		β (95%CI)	*P*	β (95%CI)	*P*	β (95%CI)	*P*
Overall study population (*n* = 402)							
Attitude							
Knowledge		0.511 (0.434,0.588)	< 0.001	0.511 (0.434,0.588)	< 0.001		
Practice							
Attitude		0.181 (0.088,0.275)	< 0.001	0.181 (0.088,0.275)	< 0.001		
Knowledge		0.663 (0.601,0.726)	< 0.001	0.571 (0.486,0.656)	< 0.001	0.093(0.043,0.142)	< 0.001
Physicians (*n* = 137)							
Attitude							
Knowledge		0.580 (0.462,0.698)	< 0.001	0.580 (0.462,0.698)	< 0.001		
Practice							
Attitude		0.189 (0.027,0.351)	0.022	0.189 (0.027,0.351)	0.022		
Knowledge		0.673 (0.571,0.775)	< 0.001	0.563 (0.413,0.713)	< 0.001	0.110 (0.014,0.206)	0.025
Nurses (*n* = 252)							
Attitude							
Knowledge		0.483 (0.383,0.584)	< 0.001	0.483 (0.383,0.584)	< 0.001		
Practice							
Attitude		0.175 (0.057,0.294)	0.004	0.175 (0.057,0.294)	0.004		
Knowledge		0.648 (0.566,0.730)	< 0.001	0.564 (0.457,0.670)	< 0.001	0.085 (0.026,0.144)	0.005

## Discussion

This cross-sectional study examined the KAP of healthcare professionals toward SCI-NP in Hunan (China). The results suggest that Hunan (China) healthcare professionals have poor knowledge, positive attitudes, and moderate practice. This study identified specific knowledge and attitude areas requiring improvements, and educational and motivational activities should be designed to improve the KAP of healthcare professionals. Improvements in knowledge and attitudes should translate into improvements in practice.

No previous studies examined the KAP of healthcare professionals toward SCI-NP. Still, some studies examined the KAP toward chronic pain and NP. Rop et al.^14^ reported that clinicians in a tertiary center had poor KAP toward the assessment and management of chronic pain. Karahan et al.^15^ reported that 80% of the nurses in the departments of physical medicine and rehabilitation, neurology, and neurosurgery had a poor knowledge of NP. In a study of urologists managing patients with prostate cancer in South Africa, only 8% were aware of the scales available to assess NP, but 74% were confident about managing NP^16^. Still, those previous studies performed in various study populations showed that the KAP toward NP is relatively poor, as shown in the present study.

In the present study, a higher job title, working in the rehabilitation department, and having experience with patients with SCI and SCI-NP were associated with higher KAP dimension scores. Those results make sense since professionals with more general experience and more experience with patients with SCI should have higher KAP, as observed regarding various health-related subjects^20–22^. Physicians and nurses showed similar KAP, but rehabilitation therapists had higher practice scores, consistent with their involvement in managing patients with SCI^23^.

Besides poor knowledge of SCI-NP in general, the present study showed that specific knowledge items would warrant further education among healthcare professionals. Indeed, knowledge about the cause of NP, the timing of the examinations for NP, the tools and scales used to evaluate NP, the tools used to evaluate anxiety and depression in SCI-NP, the need for multidisciplinary management of SCI-NP, and the non-pharmacological options for the management of NP would warrant specific teaching and training in continuing education activities. All those points are supported by guidelines^9^. In addition, a future study could examine whether trainees and residents have sufficient training on SCI-NP. Hence, improving knowledge and attitude should translate into improvements in practice. It is, therefore, essential to design interventions to improve the knowledge of healthcare professionals toward SCI-NP. Of note is that this study enrolled many participants already involved in managing SCI and SCI-NP, suggesting that they positively biased the results and hinting that the KAP of healthcare professionals would be even worse. The present study showed positive attitudes for all attitude items. Still, some practice aspects could be improved, especially regarding acquiring knowledge about SCI-NP, following the guidelines for SCI-NP, transferring knowledge to the patients, stating a long-term management plan, and specific treatment modalities for SCI-NP (e.g., respiratory control electrical stimulation, neurolysin, stem cell transplantation, meditation, hypnosis, and osteopathy)^9,24,25^.

The present study identified specific KAP items requiring education and training improvements. Improving knowledge should translate into more positive attitudes and proactive practices, as suggested by the SEM analysis and according to the KAP theoretical framework^12,13,18^. Continuing education activities could take the form of formal lectures, journal clubs, webinars, podcasts, and reading materials. Future studies should aim to design such interventions and evaluate their impact on the KAP of healthcare professionals toward SCI-NP. Improving the KAP of healthcare professionals should translate into better patient outcomes and satisfaction, but it will have to be examined in future studies.

This study had limitations. It was performed in a single region of China (Hunan). It resulted in a relatively small sample size and limited generalizability to other regions or countries with different healthcare systems, training programs, and patient populations. The sample included professionals with diverse professions, disciplines, experience, practice in leading/non-leading institutions, educational levels, etc. Although it may introduce variability, it also represents the fact that the management of SCI is multidisciplinary. The questionnaire was designed by the investigators based on local policies, guidelines, and customs, limiting the generalizability and exportability of the questionnaire. The study was cross-sectional, preventing the analysis of causality and longitudinal data. Still, a SEM was performed as a surrogate of causality to determine how the KAP dimensions influenced each other. Still, it is important to remember that such analyses provide statistically inferred causality instead of actual causality^26–28^. Because of the self-reporting of the data, all KAP studies are at risk of recall bias and social desirability bias^29,30^. Considering that the knowledge scores were poor, the influence of that bias should be relatively small. The study only examined the KAP of healthcare professionals but did not include perspectives from patients with SCI on their experiences with SCI-NP management.

In conclusion, healthcare professionals in Hunan (China) have poor knowledge, positive attitudes, and moderate practice toward SCI-NP. Specific knowledge and attitude areas requiring improvements could be identified. Hence, educational and motivational activities should be designed to improve the KAP of healthcare professionals toward SCI-NP. Based on the SEM analysis and KAP theoretical framework, improvements in knowledge and attitudes should translate into improvements in practice.

## Supplementary Information

Below is the link to the electronic supplementary material.

Supplementary Material 1

Supplementary Material 2

## Data Availability

All data generated or analysed during this study are included in this published article.
