# Supplementary material for: Knowledge, attitudes, and practices of healthcare professionals regarding neuropathic pain in spinal cord injury in Hunan, China
Source: Sci Rep. 2025 Aug 20;15:30575. doi: 10.1038/s41598-025-16252-6 (PMC12368021; doi:10.1038/s41598-025-16252-6)
Supplement: Supplementary file 1 — Supplementary Material 1 [file 41598_2025_16252_MOESM1_ESM.docx]

**Supplementary Table S1.** Spearman’s correlation analysis

| Populations and dimensions | Knowledge | Attitude | Practice |
| --- | --- | --- | --- |
| Overall study population (n=402) |  |  |  |
| Knowledge | 1.000 |  |  |
| Attitude | 0.477 (P<0.001) | 1.000 |  |
| Practice | 0.646 (P<0.001) | 0.428 (P<0.001) | 1.000 |
| Physicians (n=137) |  |  |  |
| Knowledge | 1.000 |  |  |
| Attitude | 0.527 (P<0.001) | 1.000 |  |
| Practice | 0.637 (P<0.001) | 0.468 (P<0.001) | 1.000 |
| Nurses (n=252) |  |  |  |
| Knowledge | 1.000 |  |  |
| Attitude | 0.454 (P<0.001) | 1.000 |  |
| Practice | 0.645 (P<0.001) | 0.413 (P<0.001) | 1.000 |
