# Supplementary material for: Knowledge, attitudes, and practices of healthcare professionals regarding neuropathic pain in spinal cord injury in Hunan, China
Source: Sci Rep. 2025 Aug 20;15:30575. doi: 10.1038/s41598-025-16252-6 (PMC12368021; doi:10.1038/s41598-025-16252-6)
Supplement: Supplementary file 2 — Supplementary Material 2 [file 41598_2025_16252_MOESM2_ESM.docx]

| Dear participant：  We are doctors from ** Hospital, and we sincerely invite you to participate in our research study. The purpose of this study is to understand your knowledge, attitudes, and practices regarding spinal cord injury neuropathic pain (SCI-NP) to provide a basis for developing scientific intervention strategies. This may help more people in the future by improving their understanding of the disease and their health conditions. Participation in this study is voluntary. If you agree to participate, please review the following instructions:  1. Please complete the questionnaire. There are no right or wrong answers; just provide responses based on your actual situation. If you have any questions during the process, you can ask us, and please submit the questionnaire in a timely manner once completed.  2.  This study is a simple questionnaire survey that will not harm your physical or mental health but will involve some privacy-related questions, such as your gender and age. We will keep your information strictly confidential and will not disclose it, so please fill it out with confidence.  3. As a participant, you can always inquire about information and progress related to this study. If you decide to withdraw from the study, please inform us, and your data will not be included in the research results.  Finally, we sincerely thank you for taking the time to support our scientific research!  □I have read and agree to allow the collected data to be used for scientific research.  Informed Consent Signature：  Participation date： year month day |
| --- |

| **Part I-basic information** | |
| --- | --- |
| 1. **Your current age：** | Yeas old |
| **2. your gender** | a. male  b. female |
| **3.education level：** | a. Associate degree or below b. Bachelor's degree c. Master's degree d. Medical Doctor e. Doctorate |
| **4.profession：** | a. Doctor b. Nurse |
| **5. professional title** | a. Junior b. Intermediate c. Senior d. No title |
| **6.years of work experience** | years |
| **7.** **The department you work in** | a. Rehabilitation b. Anesthesiology c. Pain Management d. Spinal Surgery e. Neurosurgery f. Other |
| **8.** **Have you ever treated or cared for patients with spinal cord injury?** | a. Yes b. No |
| **8-1.** **In the past year, the average number of spinal cord injury patients you treated/cared for per month is:** | a. Less than 1 b. 1-5 c. 6-10 d. 11-20 e. More than 20 |
| **8-2.** **Among the spinal cord injury patients you have treated or cared for, have any experienced neuropathic pain?** | a. Yes b. No |
| **8-3.** **In the past year, the average number of spinal cord injury neuropathic pain patients you treated/cared for per month is:** | a. Less than 1 b. 1-5 c. 6-10 d. 11-20 e. More than 20 |
| **9.** **What type of hospital do you work in?** | a. Public tertiary hospital b. Public secondary hospital c. Public primary hospital d. Private medical institution e. Other |
| **10.** **Is your hospital a teaching hospital?** | a. Yes b. No |
| **11.** **Your professional title:** | a. No title b. Junior c. Intermediate d. Senior |

**Part II-KNOWLEDGE**

**Please choose one of the following options for each question based on your understanding: "Understand," "Partially understand," or "Do not understand."**

| **1.** **Pain is a common complication after spinal cord injury (SCI).** | **a. understand** | **b. partially understand** | **c. do not understand** |
| --- | --- | --- | --- |
| **2.** **Neuropathic pain (NP) refers to pain caused by damage or disease affecting the somatosensory system.** | **a. understand** | **b. partially understand** | **c. do not understand** |
| **3.** **Pain screening for SCI patients should be conducted continuously during admission, hospitalization, and follow-up after discharge.** | **a. understand** | **b. partially understand** | **c. do not understand** |
| **4.** **Are you familiar with the International Spinal Cord Injury Pain Basic Data Set (ISCIPBDS)?** | **a. understand** | **b. partially understand** | **c. do not understand** |
| **5.** **All SCI patients should undergo pain assessment. The following scales can be used for NP assessment:** |  |  |  |
| **DN4 and I-DN4** | **a. TRUE** | **b. FALSE** | **c. NOT SURE** |
| **LANSS and S-LANSS** | **a. TRUE** | **b. FALSE** | **c. NOT SURE** |
| **PainDETECT** | **a. TRUE** | **b. FALSE** | **c. NOT SURE** |
| **NPQ** | **a. TRUE** | **b. FALSE** | **c. NOT SURE** |
| **ID pain** | **a. TRUE** | **b. FALSE** | **c. NOT SURE** |
| **6.** **Electrophysiological tests play an important role in the diagnosis of NP. The following tests are helpful for NP assessment:** | **a. TRUE** | **b. FALSE** | **c. NOT SURE** |
| **Nerve conduction studies** | **a. TRUE** | **b. FALSE** | **c. NOT SURE** |
| **F-waves and H-reflexes** | **a. TRUE** | **b. FALSE** | **c. NOT SURE** |
| **Quantitative sensory testing** | **a. TRUE** | **b. FALSE** | **c. NOT SURE** |
| **Skin sympathetic response** | **a. TRUE** | **b. FALSE** | **c. NOT SURE** |
| **7.** **The following imaging studies are helpful for diagnosing NP:** |  |  |  |
| **CT** | **a. TRUE** | **b. FALSE** | **c. NOT SURE** |
| **MRI** | **a. TRUE** | **b. FALSE** | **c. NOT SURE** |
| **fMRI** | **a. TRUE** | **b. FALSE** | **c. NOT SURE** |
| **PET-CT** | **a. TRUE** | **b. FALSE** | **c. NOT SURE** |
| **8.** **Pain is related to psychological states such as anxiety and depression. PHQ-9 and GAD-7 are commonly used for psychological assessment in NP patients.** | **a. understand** | **b. partially understand** | **c. do not understand** |
| **9.** **Management of NP requires a principle of comprehensive management and multidisciplinary collaboration.** | **a. understand** | **b. partially understand** | **c. do not understand** |
| **10.** **First-line medications for NP include:** |  |  |  |
| **Anticonvulsants** | **a. TRUE** | **b. FALSE** | **c. NOT SURE** |
| **Antidepressants** | **a. TRUE** | **b. FALSE** | **c. NOT SURE** |
| **Opioids** | **a. TRUE** | **b. FALSE** | **c. NOT SURE** |
| **Topical analgesics** | **a. TRUE** | **b. FALSE** | **c. NOT SURE** |
| **11.** **Minimally invasive interventional treatments for NP include:** |  |  |  |
| **Nerve blocks** | **a. TRUE** | **b. FALSE** | **c. NOT SURE** |
| **Pulsed radiofrequency** | **a. TRUE** | **b. FALSE** | **c. NOT SURE** |
| **Neurolysis** | **a. TRUE** | **b. FALSE** | **c. NOT SURE** |
| **12.** **Neuromodulation treatments for NP include neurostimulation and intrathecal drug delivery.** | **a. understand** | **b. partially understand** | **c. do not understand** |
| **13.** **Surgical treatments for NP mainly involve nerve decompression and neurodestruction.** | **a. understand** | **b. partially understand** | **c. do not understand** |
| **14.** **Are you familiar with the following NP treatments:** |  |  |  |
| **Psychotherapy** | **a. understand** | **b. partially understand** | **c. do not understand** |
| **Rehabilitation therapy** | **a. understand** | **b. partially understand** | **c. do not understand** |
| **Physical therapy** | **a. understand** | **b. partially understand** | **c. do not understand** |
| **Gene therapy** | **a. understand** | **b. partially understand** | **c. do not understand** |

**Part-III attitudes**

**Please select one option from "Strongly agree" to "Strongly disagree" based on your agreement with the statements.**

| **1.** **NP has a significant impact on SCI patients' emotions, quality of life, and rehabilitation.** | **a. strongly agree** | **b. agree** | **c. neutral** | **d. disagree** | **e. strongly disagree** |
| --- | --- | --- | --- | --- | --- |
| **2.** **Early detection and treatment of NP are important for the rehabilitation of SCI patients.** | **a. strongly agree** | **b. agree** | **c. neutral** | **d. disagree** | **e. strongly disagree** |
| **3.** **When assessing NP, the patient's concerns, expectations, and needs should be considered.** | **a. strongly agree** | **b. agree** | **c. neutral** | **d. disagree** | **e. strongly disagree** |
| **4.** **The assessment and management of SCI-NP require multidisciplinary collaboration.** | **a. strongly agree** | **b. agree** | **c. neutral** | **d. disagree** | **e. strongly disagree** |
| **5.** **Self-management by patients plays an important role in the management of SCI-NP.** | **a. strongly agree** | **b. agree** | **c. neutral** | **d. disagree** | **e. strongly disagree** |
| **6.** **Treatment of SCI-NP should consider both pharmacological and non-pharmacological treatment options.** | **a. strongly agree** | **b. agree** | **c. neutral** | **d. disagree** | **e. strongly disagree** |
| **7.** **Management of SCI-NP should continue after the patient is discharged.** | **a. strongly agree** | **b. agree** | **c. neutral** | **d. disagree** | **e. strongly disagree** |
| **8.** **Before the discharge of SCI-NP patients, a comprehensive follow-up rehabilitation plan should be developed, and self-management skills should be taught to the patients.** | **a. strongly agree** | **b. agree** | **c. neutral** | **d. disagree** | **e. strongly disagree** |

**Part IV- practice**

**Please select the option that best describes your actions in the corresponding situations.**

| **1.** **I actively learn about the management of SCI-NP.** | **a. always** | **b. often** | **c. sometimes** | **d. rarely** | **e. never** |
| --- | --- | --- | --- | --- | --- |
| **2.** **I actively learn about the latest developments in SCI-NP or NP research.** | **a. always** | **b. often** | **c. sometimes** | **d. rarely** | **e. never** |
| **3.** **During the management of SCI patients, I follow guidelines for pain assessment.** | **a. always** | **b. often** | **c. sometimes** | **d. rarely** | **e. never** |
| **4.** **When managing SCI-NP patients, I first provide education to the patients.** | **a. always** | **b. often** | **c. sometimes** | **d. rarely** | **e. never** |
| **5.** **When using medication for treatment, I strictly adhere to guidelines or recommendations.** | **a. always** | **b. often** | **c. sometimes** | **d. rarely** | **e. never** |
| **6.** **Before SCI-NP patients are discharged, I provide them with a long-term rehabilitation plan.** | **a. always** | **b. often** | **c. sometimes** | **d. rarely** | **e. never** |
| **7.** **Before SCI-NP patients are discharged, I provide education on self-management.** | **a. always** | **b. often** | **c. sometimes** | **d. rarely** | **e. never** |
| **8.** **The following are treatments not yet explicitly recommended by guidelines but may be effective for SCI-NP. When patients meet the treatment criteria, how willing are you to recommend these treatments?** |  |  |  |  |  |
| **Cognitive Behavioral Therapy** | **a. strongly recommend** | **b. recommend** | **c. neutral** | **d. do not recommend** | **e. not heard of it** |
| **Hyperbaric Oxygen Therapy** | **a. strongly recommend** | **b. recommend** | **c. neutral** | **d. do not recommend** | **e. not heard of it** |
| **Anti-inflammatory Diet** | **a. strongly recommend** | **b. recommend** | **c. neutral** | **d. do not recommend** | **e. not heard of it** |
| **Respiratory Control Electrical Stimulation** | **a. strongly recommend** | **b. recommend** | **c. neutral** | **d. do not recommend** | **e. not heard of it** |
| **Neurolysin** | **a. strongly recommend** | **b. recommend** | **c. neutral** | **d. do not recommend** | **e. not heard of it** |
| **Autologous Mesenchymal Stem Cell Transplantation** | **a. strongly recommend** | **b. recommend** | **c. neutral** | **d. do not recommend** | **e. not heard of it** |
| **Meditation** | **a. strongly recommend** | **b. recommend** | **c. neutral** | **d. do not recommend** | **e. not heard of it** |
| **Hypnosis** | **a. strongly recommend** | **b. recommend** | **c. neutral** | **d. do not recommend** | **e. not heard of it** |
| **Acupuncture** | **a. strongly recommend** | **b. recommend** | **c. neutral** | **d. do not recommend** | **e. not heard of it** |
| **Exercise** | **a. strongly recommend** | **b. recommend** | **c. neutral** | **d. do not recommend** | **e. not heard of it** |
| **Osteopathy** | **a. strongly recommend** | **b. recommend** | **c. neutral** | **d. do not recommend** | **e. not heard of it** |

| **Thank you for filling out our questionnaire! The information you have provided is very valuable for our future work.**  **Thank you for filling out our questionnaire！**  If you have any comments or suggestions about this research study, we would be honored to hear your feedback.  Comments and Suggestions： （Optional） |
| --- |
